# Exertion-Induced Spontaneous Pneumomediastinum in a Healthy Young Male: A Case Report

**DOI:** 10.7759/cureus.97130

**Published:** 2025-11-17

**Authors:** Muhammad Talha Zahid, Samrah Afzal

**Affiliations:** 1 Cardiology, King's Mill Hospital, Sutton-in-Ashfield, GBR; 2 Emergency Medicine, King's Mill Hospital, Sutton-in-Ashfield, GBR

**Keywords:** alveolar rupture, oesophageal perforation, spontaneous pneumomediastinum (spm), surgical emphysema, valsalva maneuver, vaping

## Abstract

Spontaneous pneumomediastinum (SPM) is an uncommon, self-limiting condition that usually resolves with supportive management. In this condition, air enters the central chest cavity due to increased intra-alveolar pressure, causing alveolar rupture. It is likely seen in young adults who are usually fit and healthy without any underlying disease or pathology. It is a benign condition that can present as chest pain, breathlessness, difficulty swallowing, neck and chest emphysema, and, rarely, hoarseness of voice or odynophagia. We report the case of a 19-year-old male who presented to the emergency department with deep chest pain and bubbles under the skin. He was a non-smoker but had a history of vaping for two years. On examination, he had surgical emphysema in the neck and chest. In this case, the exertional strain from a new gym exercise is likely to have contributed to the alveolar rupture. Chest radiography and computed tomography (CT) of the thorax showed air in the mediastinum. The patient was managed conservatively, supported with analgesia and rest. The symptoms and surgical emphysema resolved after two days, and he was discharged without any complications.

## Introduction

Pneumomediastinum is a rare condition defined by the presence of air in the mediastinum. In the absence of traumatic injury, iatrogenic injury, or clear etiology, it is called spontaneous pneumomediastinum (SPM) [1]. It is an uncommon clinical entity that most frequently affects young, healthy males without underlying pulmonary disease [1,2]. The condition can occur after activities that cause a sudden rise in intra-alveolar pressure, such as vigorous coughing, performing a Valsalva maneuver, vomiting, strenuous physical activity, or inhaling substances like vape aerosols or recreational drugs [3,4]. The principal mechanism, described as the Macklin effect, involves alveolar rupture secondary to an abrupt rise in intra-alveolar pressure, leading to air dissection along bronchovascular sheaths toward the mediastinum [5].

Patients typically present with sudden onset retrosternal or pleuritic chest pain, dyspnoea, neck swelling, or subcutaneous crepitus due to air tracking into the soft tissues. Although alarming in presentation, SPM is generally benign and self-limiting once life-threatening secondary causes, particularly oesophageal perforation, have been excluded [2,6]. Chest radiography, computed tomography (CT) of the thorax, and contrast swallow fluoroscopy are among the diagnostic modalities. Most cases resolve with conservative management without any complications.

## Case presentation

A 19-year-old male with no past medical or surgical history presented in the emergency department with chest pain and bubbles under the skin. He woke up with these symptoms one day before presentation. Chest pain was non-radiating, deep, central, and 3/10 in intensity, which aggravated on bending over. There was no history of trauma, cough, shortness of breath, hoarseness of voice, difficulty in eating or swallowing, vomiting, or fever. He had no complaint of choking, wheezing, or aspiration of food. The bubbles under the skin were limited to both sides of the neck, the upper chest, and the tops of the shoulders. He used to vape but no cigarette smoking, as well as no known allergy. Notably, the day before symptom onset, he had engaged in a new, strenuous weightlifting routine at the gym.

Physical examination revealed non-tender, palpable crepitus in the subcutaneous tissues of the bilateral neck, upper chest, and shoulders, with a clear chest on auscultation. Upper airway assessment was unremarkable, and he demonstrated stable vital signs: National Early Warning Score (NEWS) of 0, respiratory rate 20 breaths per minute, oxygen saturation 100% on ambient air, heart rate 82 beats per minute, blood pressure 147/80 mmHg, and temperature 36.2°C. Laboratory investigations were within normal limits with the exception of a mildly elevated C-reactive protein of 13 mg/L, as shown in Table 1.

**Table 1:** Laboratory investigations. PT: prothrombin time; INR: international normalized ratio; eGFR (by EPI): estimated glomerular filtration rate (Chronic Kidney Disease - Epidemiology Collaboration)

Parameter	Result	Reference Range	Units
C-reactive Protein	13	0-5	mg/L
Haemoglobin	159	130-170	g/L
White Cell Count	7.7	4-10	×10^9^/L
Platelet Count	287	150-410	×10^9^/L
Red Cell Count	5.31	4.5-5.5	×10^12^/L
Haematocrit	0.464	0.40-0.50	L/L
Mean Corpuscular Volume (MCV)	87	83-101	fL
Mean Corpuscular Haemoglobin (MCH)	29.9	27-32	pg
Mean Corpuscular Haemoglobin Concentration (MCHC)	343	315-345	g/L
Neutrophils	4.5	2.0-7.0	×10^9^/L
Lymphocytes	2.1	1.0-3.0	×10^9^/L
Monocytes	0.8	0.2-1.0	×10^9^/L
Eosinophils	0.3	0.0-0.5	×10^9^/L
Basophils	0.1	0.0-0.1	×10^9^/L
Nucleated RBCs	0.0	0.0-0.1	×10^9^/L
PT Ratio/INR	1.0	-	Ratio
Activated Partial Thromboplastin Time (APTT)	31	25-37	sec
PT (non-anticoagulated)	11.4	9.7-13.7	sec
Sodium	142	133-146	mmol/L
Potassium	3.9	3.5-5.3	mmol/L
Urea	5.5	2.5-7.8	mmol/L
Creatinine	88	70-120	µmol/L
eGFR (by EPI)	>90	-	mL/min/1.73 m^2^

**Figure 1:**
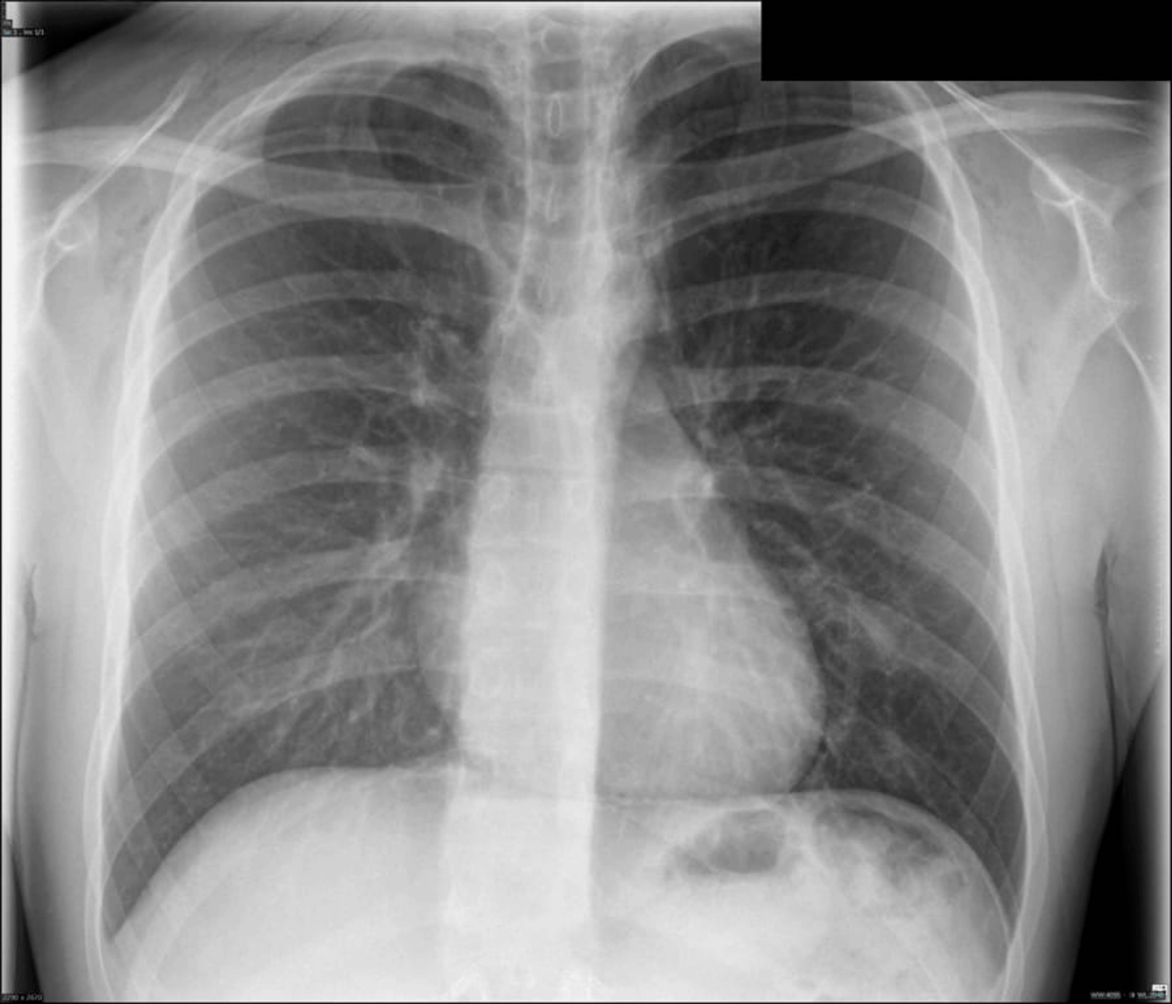
Subcutaneous emphysema in the lower neck and shoulders, with radiographic features of pneumomediastinum on the chest X-ray.

**Figure 2:**
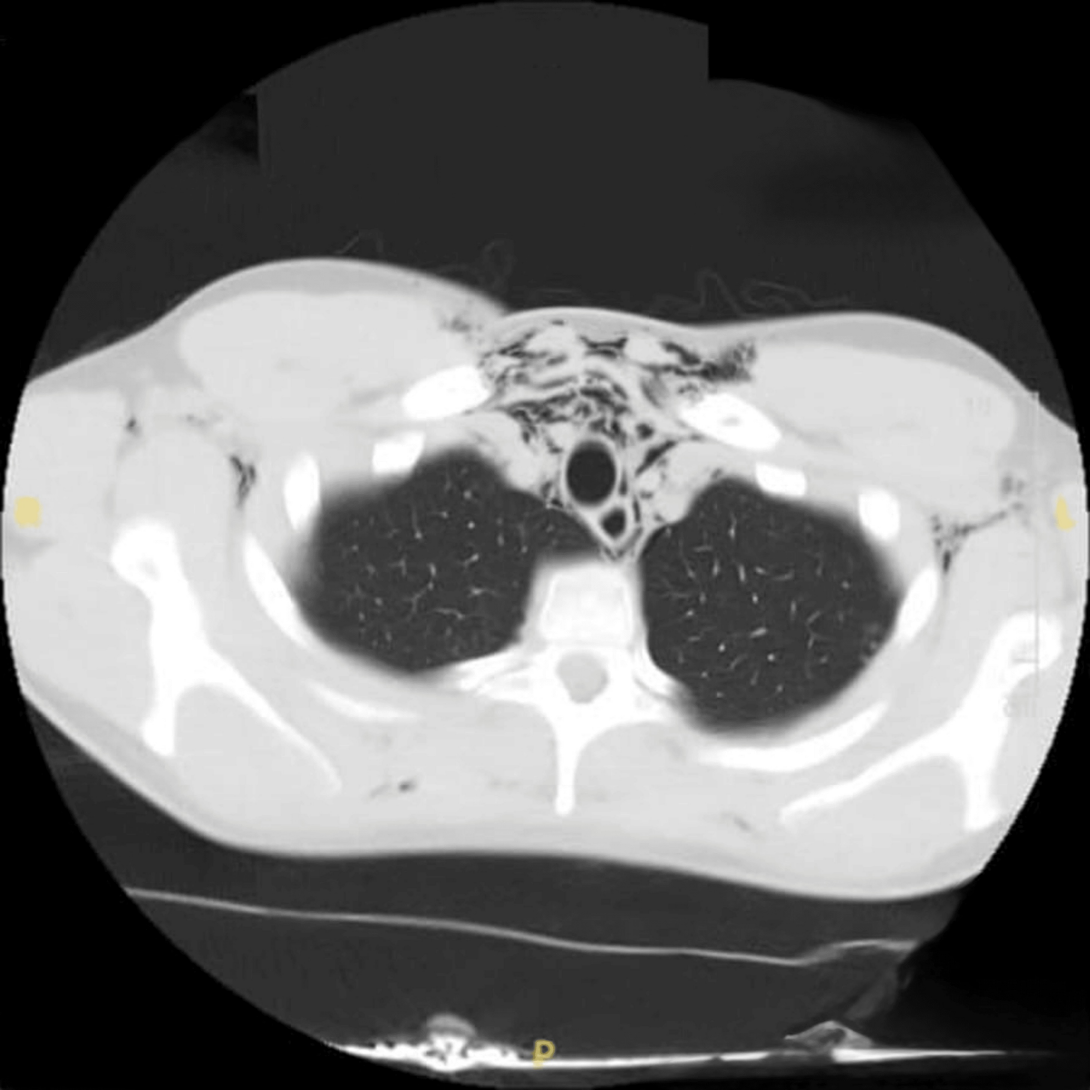
Computed tomography (CT) of the thorax showing moderate pneumomediastinum with surgical emphysema.

**Figure 3:**
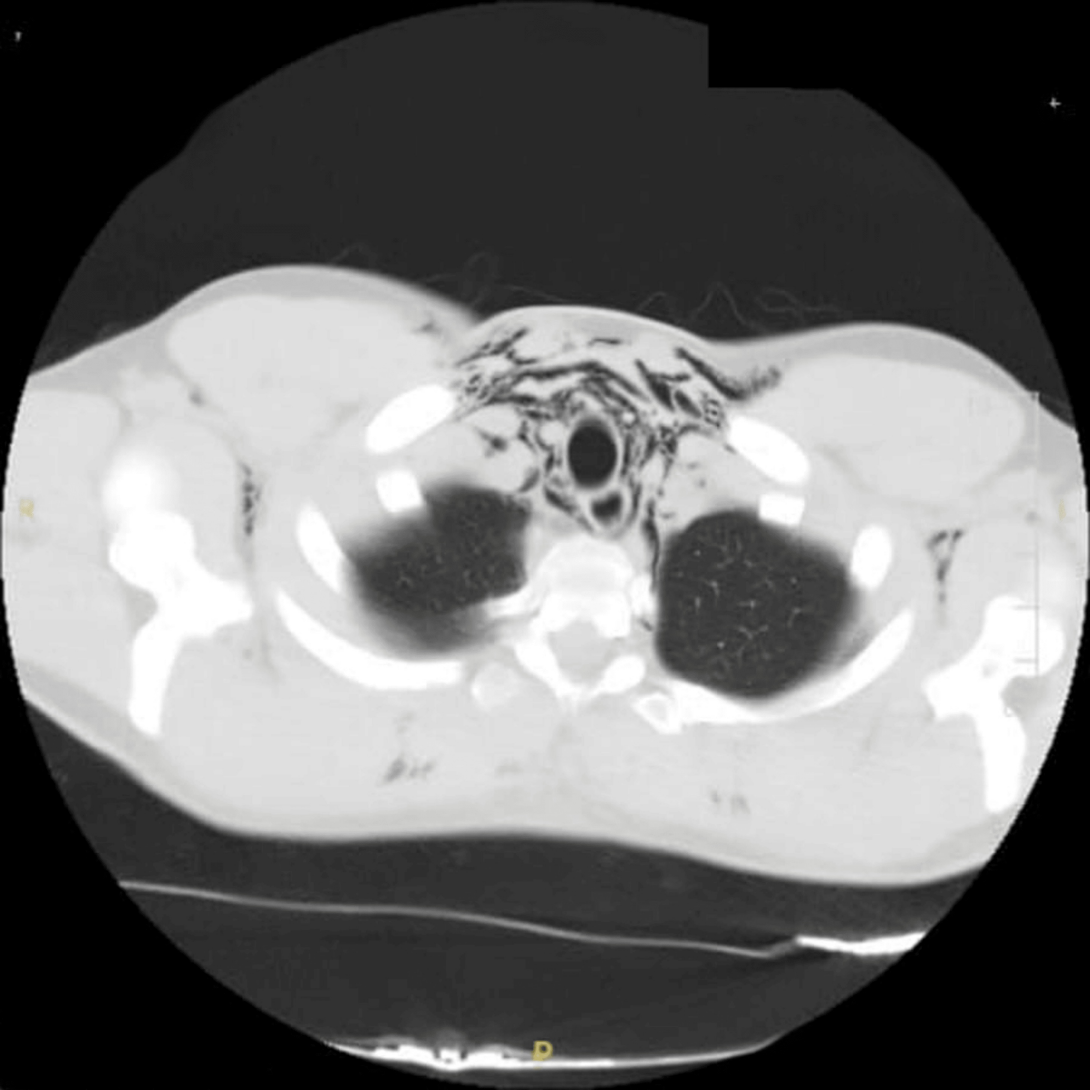
Computed tomography (CT) of the thorax showing air within the mediastinum.

Upon presentation, a chest radiograph was performed, which showed subcutaneous emphysema in the lower neck/shoulders and features of pneumomediastinum, as shown in Figure 1. This was followed by a CT of the thorax that showed moderate pneumomediastinum with surgical emphysema tracking into pretracheal and paratracheal spaces, extending into the neck with no radiologically apparent cause, i.e., no oesophageal rupture, no pneumothorax, no bony abnormality or lung’s pathology (Figures 2-3). In the absence of alternative etiologies, exertional strain associated with weightlifting was considered the likely precipitant.

The patient was kept nil by mouth and managed with intravenous fluids while a water-soluble contrast swallow study was obtained. This excluded any oesophageal or pharyngeal perforation. During hospitalization, the patient remained clinically and hemodynamically stable. Following multidisciplinary discussion with the surgical team, oral intake was reintroduced, and he was admitted to the medical ward for observation. His chest pain and subcutaneous emphysema improved markedly within 24 hours. After two days of inpatient monitoring and a return-to-baseline examination, he was deemed medically fit for discharge. He was counseled to discontinue vaping and refrain from heavy lifting. A follow-up chest radiograph was scheduled for three to four weeks to confirm radiological resolution prior to his planned air travel the following month.

## Discussion

SPM is a rare clinical condition, with an incidence ranging from 1 in 7,000 to 1 in 45,000 in the general population. It is characterized by pneumatisation of the mediastinal cavity without precipitating trauma, surgery, or obvious underlying disease. It is most often described in young, healthy males and usually follows a benign, self-limiting course once serious secondary causes have been excluded [1,2]. The case presented here involved a 19-year-old male who developed chest pain and subcutaneous emphysema after performing a new gym routine, consistent with exertion-induced SPM.

The pathogenesis of SPM is explained by the Macklin effect, first described by Macklin and Macklin in 1944 [5]. When intrathoracic pressure rises suddenly, such as during a Valsalva manoeuvre, forceful coughing, or intense physical exertion, alveoli may rupture. The escaped air then tracks along the peribronchial and perivascular connective tissues toward the mediastinum. This mechanism accounts for the absence of trauma or direct injury in many cases of SPM [5,7]. In this case, the strain of weightlifting likely caused a transient increase in alveolar pressure sufficient to rupture alveolar walls. The patient’s history of vaping might have further contributed, as deep inhalation and barotrauma from vaping have been associated with similar presentations [8]. Deep and forceful inhalation during vaping raises intra-alveolar and intrathoracic pressure, which can exceed the tensile strength of alveolar walls, leading to rupture and air leak.

Clinically, patients with SPM often report acute retrosternal chest pain, sometimes radiating to the neck or back, and may notice swelling or crepitus in the neck and upper chest [3,4]. Dyspnoea, dysphagia, or odynophagia can also occur, though most remain hemodynamically stable [2,6]. These symptoms closely matched those observed in the present case. Although the physical findings can appear alarming, SPM typically resolves with conservative therapy. Nonetheless, it is essential to differentiate SPM from life-threatening causes of pneumomediastinum, especially oesophageal perforation, which carries high morbidity and mortality if untreated [9].

Chest radiography is typically the first diagnostic test performed; however, thoracic CT offers greater sensitivity and helps rule out pneumothorax, underlying lung disease, or esophageal injury. A water-soluble contrast swallow is recommended when there is any suspicion of oesophageal perforation, particularly in patients presenting with vomiting or a history suggestive of Boerhaave’s syndrome [9]. In the present case, both CT and contrast swallow confirmed the absence of an oesophageal leak, validating a conservative management approach.

Management of SPM primarily aims to relieve symptoms and to ensure careful monitoring. The cornerstone of treatment includes providing supplemental oxygen, ensuring bed rest, and administering appropriate analgesia. The patient in this case improved with supportive measures alone, and subcutaneous emphysema resolved within 48 hours, consistent with prior studies reporting favourable short-term outcomes [2,6,7].

Recurrence of SPM is rare; however, it may occur when underlying predisposing factors remain unresolved. Patients should therefore be advised to avoid heavy lifting, strenuous exertion, and inhalational activities such as vaping or recreational drug use, as they come with risks [8]. A follow-up chest radiograph several weeks after initial imaging is advisable to ensure complete resolution before resuming exercise or air travel.

This case highlights the necessity of considering SPM as a potential diagnosis in young adults who present with acute chest pain and subcutaneous emphysema, despite its low incidence. From a clinical perspective, the implications are twofold: first, prompt identification and thorough imaging are essential to exclude life-threatening etiologies such as esophageal perforation or pulmonary pathology, guiding safe and effective patient management. Second, the benign and self-limited nature of SPM, once secondary causes are ruled out, supports a conservative therapeutic approach that minimizes unnecessary interventions and hospital resource utilization. By maintaining a high index of suspicion for SPM and employing evidence-based diagnostic strategies, clinicians can reduce diagnostic delays, prevent mismanagement, and enhance patient outcomes.

## Conclusions

SPM is a rare but important cause of chest pain in healthy young adults. In individuals with a history of vaping, sudden exertion can trigger alveolar rupture via the Macklin effect. Prompt imaging is essential to exclude life-threatening causes, such as oesophageal perforation. Once serious differentials are ruled out, conservative management is usually sufficient, with excellent outcomes. Follow-up imaging can ensure complete resolution and help prevent recurrence.
